# Immunotherapy Platform That Conjugates Antigen to Complement C3-Targeted Liposomes Induces a Robust Adaptive Immune Response

**DOI:** 10.3390/ijms26114985

**Published:** 2025-05-22

**Authors:** R. G. Barber, Steven Cherry, Sydney Stephens, Kristine Mann, Holly A. Martinson, Max Kullberg

**Affiliations:** 1 WWAMI School of Medical Education, University of Alaska Anchorage, 3211 Providence Drive, Anchorage, AK 99508, USA; rgbarber@alaska.edu (R.G.B.); srstephens2@alaska.edu (S.S.);; 2 Department of Biological Sciences, University of Alaska Anchorage, 3211 Providence Drive, Anchorage, AK 99508, USA; kmann1@alaska.edu

**Keywords:** cancer, toll-like receptor agonist, antigen presenting cells, immune modulation, complement C3 targeting, liposomal delivery

## Abstract

The activation of immunosuppressed antigen-presenting cells (APCs) in the tumor microenvironment is a key goal in modern cancer immunotherapy. Our laboratory utilizes a liposome-based immunotherapy platform that binds endogenous complement to deliver antigen, adjuvant, and therapeutic agents to APCs in vivo. The liposomes contain external linker groups, which readily bind complement protein C3, and mediate liposomal uptake via complement receptor 3 into APCs. To test the ability of a model antigen to bind to these external linker groups on C3-liposomes and elicit a robust adaptive immune response, we conjugated a modified ovalbumin peptide (OVA-C) to the liposomes and incorporated a toll-like receptor (TLR) 4 agonist, monophosphoryl lipid A (MPLA), in the liposomal membrane. Adaptive immune responses from C57BL/6 mice were analyzed by ELISA and ELISpot. Mice vaccinated with OVA-C liposomes elicited significantly greater humoral and cellular adaptive responses relative to controls. Furthermore, female mice vaccinated with MPLA + OVA-C liposomes produced significantly more IgG antibodies than males vaccinated with the same liposomes. In conclusion, antigen binding on the exterior of C3-liposomes markedly improves antigen loading efficiency and still allows for complement C3-targeted delivery to APCs. These data demonstrate the initiation of a robust cellular and humoral immune response using a new liposomal delivery platform.

## 1. Introduction

The ability to avoid the immune response is considered a hallmark of cancer and is recognized as a necessary step for tumor progression [1,2]. Cancer cells evade immune detection by a variety of methods, including the downregulation of MHC I, the upregulation of PD-L1, and the release of regulatory cytokines, which lead to the suppression of immune cells in the tumor microenvironment [3]. The goal of cancer immunotherapy is to augment or reactivate the patient’s immune response and to enhance the ability of the immune system to recognize tumor antigens and eliminate cancer cells [4].

The adaptive immune response is primarily carried out by cytotoxic T lymphocytes (CTLs) and B cells. Both are antigen-specific effector cells that require antigen presentation and activation by antigen-presenting cells (APCs), such as macrophages and dendritic cells (DCs) [5]. APCs bridge the innate immune and adaptive immune responses by processing available antigens from their environment and displaying them to T cells and B cells, along with co-stimulatory factors [5,6]. These antigens are often obtained through the coating of pathogens with complement and subsequent internalization into APCs mediated by complement receptor 3. APCs process the pathogen and display antigens to effector cells through MHC I and MHC II, while danger-associated molecular patterns from the pathogen lead to APC activation. APCs are often immunosuppressed in cancer patients due to the lower expression of MHC II, CD80/86, and mTORC1 and the increased production of immunosuppressive cytokines such as IL-6 and IL-10 [7]. APC suppression plays a central role in the inhibition of the adaptive immune response and in the reduced efficacy of applied immunotherapies [8]. To overcome this suppression, there exists a need for treatments that target immunosuppressed APCs for reactivation while simultaneously delivering antigen.

Our laboratory has developed a liposome-based delivery system that utilizes part of the complement cascade to target APCs and simultaneously deliver antigen and adjuvants [9]. The liposomes contain a lipid with a linker group, orthopyridyl disulfide (OPSS), on the exterior of the liposomes, where it forms covalent disulfide bonds with other molecules under physiological conditions. In serum, the OPSS linker groups readily bind to complement protein C3 and mediate the delivery of the liposomes to immune cells that express C3b receptors. This set of immune cells includes all three APC subtypes: B cells, macrophages, and DCs [10]. Prior research in our laboratory using model tumor antigen ovalbumin (OVA) has shown the ability of this system to target APCs, elicit a significant increase in activated antigen-specific T cells, and suppress growth in established A20-OVA tumors [11]. One drawback of using OVA peptide as a mock antigen is that it is derived from a different species than the mice being vaccinated and can result in robust immune responses that may not necessarily be replicated when tumor antigens are substituted into the system. However, it is a well-characterized antigen and could be a good indicator of how our liposomal delivery system would perform if used to deliver foreign peptides from viruses and bacteria.

Previous iterations of the C3-liposome system utilized a passive method for encapsulating hydrophilic antigens during extrusion [11,12,13]. This process could be challenging to scale up to pharmaceutically relevant quantities, given the costs associated with the low loading efficiency of the antigen and the methods required for separating liposomes from unencapsulated antigens. To improve upon our method, we have explored the possibility of conjugating peptides directly to the exterior of the liposomes through the same OPSS groups used to bind complement C3 after the liposomes have been formed. To evaluate the efficacy of this system, liposomes were covalently linked to an ovalbumin-derived peptide with an additional four amino acids (-GGGC) at the C-terminus (OVA-C), a strategy that has been successful for attaching peptides to nanoparticles using SMPH as an intermediary [14]. At physiological pH, the OPSS groups bind to the available cysteine residue on the OVA-C peptides, conjugating OVA-C to the exterior of the pre-formed liposomes and leaving sufficient OPSS groups available for the subsequent binding of endogenous complement C3. Following vaccination with OVA-C liposomes, mice were evaluated for humoral and cellular immune responses. We demonstrate that targeted antigen delivery to APCs utilizing OVA-C liposomes enhances the adaptive T cell and antibody response in mice. Our study suggests this peptide + C3-liposome conjugation method may allow for an efficient delivery system that could be utilized in personalized cancer therapies.

## 2. Results

### 2.1. Evaluation of Peptide and Complement Binding Capacity of OPSS-Liposomes

**Figure 1:**
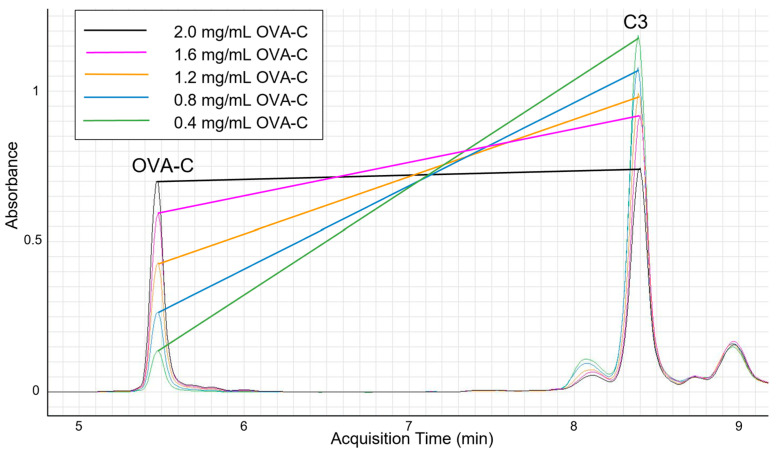
HPLC analysis of complement C3 and OVA-C bound to liposomes.

To evaluate the binding capacity of OPSS-liposomes for both peptide and complement C3, liposomes were incubated overnight with OVA-C peptide before exposure to C3+ murine serum. We hypothesized that the peptide concentration would need to be limited to ensure that sufficient OPSS groups would be available for the subsequent binding of complement C3. To test this hypothesis, liposomes were first incubated overnight with various concentrations of OVA-C peptide to ensure complete peptide conjugation to available OPSS groups. The liposomes were subsequently exposed to C3+-containing serum for 30 min in vitro, allowing complement C3 to bind to any remaining free OPSS groups. HPLC analysis confirmed that as the amount of conjugated OVA-C peptide increased, the amount of C3 bound to the liposomes subsequently decreased (Figure 1). These findings demonstrate that an antigenic peptide can be conjugated to OPSS-liposomes while still permitting complement C3 attachment.

Aliquots of 200 μL of OPSS-liposomes were incubated overnight with 25 μL of varying concentrations of OVA-C (noted in the inset above), followed by incubation with C3+ murine serum, before being run over a CL4B size exclusion column. After the CL4B column, dithiothreitol was used as a reducing agent to break the disulfide bonds between OPSS and either C3 or the terminal cysteine residue on the OVA-C peptide. Liposomes of uniform size were collected, and each sample containing varying amounts of OVA-C was analyzed by HPLC. OVA-C retention time was 5.48 min, while the primary complement C3 peak occurred at approximately 8.39 min. Our analysis shows an inverse relationship between the amount of OVA-C bound to the liposomes and the amount of C3 bound to the same liposomal pool.

### 2.2. Biodistribution of OVA-C Liposomes in Blood and Spleen

To assess whether direct antigen conjugation to the OPSS-liposomal surface still allows for the uptake of OVA-OPPS-liposomes (OVA-C) by CD11b+ myeloid cells, we conducted an in vivo biodistribution study. Complement receptor 3 (CD11b) is one of several complement receptors that bind to complement C3b and mediate myeloid cell uptake. Our previous work demonstrated that the conjugation of C3b to exposed OPSS groups on liposomes enhances their uptake by myeloid cells [9,10].

**Figure 2:**
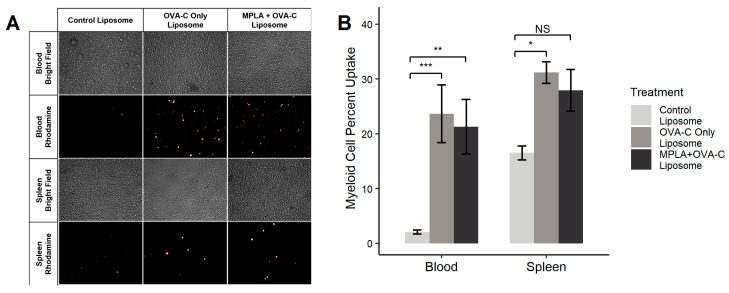
OVA-C liposome biodistribution. Liposomes without MPLA or OPSS groups (control liposome), OPSS-liposomes incubated with OVA-C (OVA-C-only liposome), or OPSS + MPLA liposomes incubated with OVA-C (MPLA + OVA-C liposome) were intravenously injected into mice via the tail vein. All liposomes contained rhodamine-labeled phospholipids. After 1 h, mice were sacrificed, and blood and spleens were collected and processed for (**A**) fluorescent microscopy imaging using a 20× objective and (**B**) flow cytometry analysis of liposome uptake in myeloid cells (CD11b+). Images in (**A**) are representative of 3 mice per group. In flow analysis (**B**), * *p* < 0.05, ** *p* < 0.01, and *** *p* < 0.005; NS = not significant.

Nine mice received a tail vein injection of MPLA + OVA-C liposomes, OVA-C-only liposomes, or control liposomes lacking OPSS or MPLA. After one hour, blood and spleen samples were collected and analyzed via fluorescent microscopy (Figure 2A) and flow cytometry (Figure 2B). In the blood, control liposomes were taken up by 2.08% ± 0.3% of myeloid cells, while OVA-C-only and MPLA + OVA-C liposomes were taken up by 23.7% ± 5.2% and 21.3% ± 4.9% of myeloid cells (*p* = 0.002 and 0.006 compared to the control), respectively. In the spleen, 16.5% ± 1.25% of myeloid cells took up control liposomes, while OVA-C-only and MPLA + OVA-C liposomes were taken up by 31.2% ± 2.0% and 27.9% ± 3.8% of myeloid splenocytes (*p* = 0.04 and 0.149 compared to the control), respectively. These findings support our prior research, confirming that OPSS-liposomes enhance myeloid cell uptake in the blood and in the spleen.

### 2.3. Adaptive Immune Response to OVA-C Liposomes

To assess the adaptive immune response to OVA-C liposomes, C57BL/6 mice were vaccinated twice over a two-week period on day 0 and day 7 with MPLA + OVA-C liposomes, OVA-C-only liposomes, or free OVA-C peptide. The adaptive immune response was evaluated using ELISA to measure serum IgG anti-OVA-C concentrations and ELISpot to examine the IFN-gamma T cell response. An HPLC analysis of liposomes used for vaccination showed 24.54 μg of OVA-C in the MPLA + OVA-C liposomes and 27.24 μg in the OVA-C-only liposomes, indicating an average OVA-C conjugation efficiency of 84.6% of the original 30 μg of OVA-C incubated with the liposomes. This is a considerable increase over passive encapsulation, which has a loading efficiency of approximately 5% [12]. Liposomes and free OVA-C were normalized to an OVA-C concentration of 35 μg/mL prior to vaccination.

#### 2.3.1. Antibody Immune Response to Vaccination

**Figure 3:**
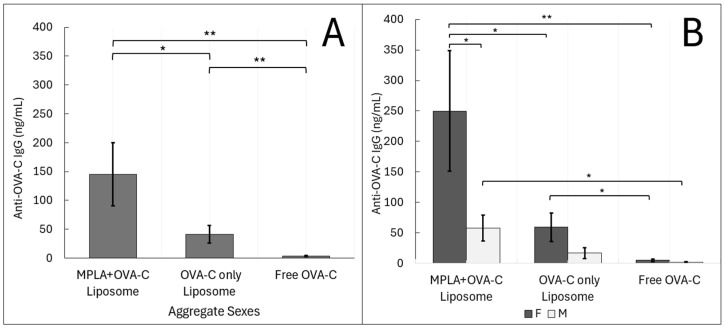
ELISA Analysis: Liposomal delivery of OVA-C leads to an IgG adaptive humoral immune response. Mice were vaccinated with MPLA + OVA-C liposomes, OVA-C-only liposomes, or free OVA-C peptide, and tissue was collected after two weeks. (**A**) Collected serum samples were analyzed via ELISA for the concentration of anti-OVA-C IgG present. (**B**) Data were further analyzed by sex. The data are expressed as the mean serum IgG concentrations (ng/mL ± standard error). The experiment was performed with 6 cohorts; *n* = 11–14 per vaccination group, as described in the Materials and Methods section (* *p* < 0.05) (** *p* < 0.01).

Sera from vaccinated mice were analyzed for anti-OVA-C IgG antibodies using a modified peptide-based ELISA (Figure 3A). Mice vaccinated with MPLA + OVA-C liposomes (aggregate sex) exhibited an average antibody concentration of 145.2 ± 57.2 ng/mL (mean ± standard error), nearly 50-fold greater than that of mice vaccinated with free OVA-C (3.5 ± 0.9 ng/mL) (*p* = 0.0002). Mice vaccinated with OVA-C-only liposomes also showed a higher IgG concentration (41.3 ± 15.6 ng/mL) than that of free OVA-C mice (*p* = 0.003). However, OVA-C-only liposomes had a significantly lower IgG concentration than in mice vaccinated with MPLA + OVA-C liposomes (*p* = 0.028).

When groups were analyzed by sex, some of these differences lost significance (Figure 3B). Female mice vaccinated with MPLA + OVA-C liposomes had approximately 5-fold higher serum antibody concentrations (249.9 ± 110.3 ng/mL) compared to those receiving OVA-C-only liposomes (59.4 ± 25.1 ng/mL) (*p* = 0.017) and approximately 46-fold higher compared to free OVA-C (5.4 ± 1.52 ng/mL) (*p* = 0.0051). In female mice, OVA-C-only liposome vaccination resulted in a roughly 5-fold higher antibody concentration than free OVA-C (*p* = 0.024).

In contrast, male mice had only a 20-fold higher antibody concentration when vaccinated with MPLA + OVA-C liposomes (58.0 ± 23.4 ng/mL) compared to free OVA-C mice (1.5 ± 0.6 ng/mL) (*p* = 0.0129). Male mice vaccinated with OVA-C-only liposomes exhibited an average antibody concentration of 17.0 ± 10.0 ng/mL, not significantly different from any other male treatment group. Notably, sex-based differences were only significant in the MPLA + OVA-C liposome vaccinated group, where female mice had a nearly 5-fold greater anti-OVA-C IgG concentration than males (*p* = 0.031).

Overall, female mice demonstrated greater responsiveness across all vaccination groups, with significant differences observed between each group. In contrast, male mice had generally lower immune responses and required the addition of MPLA to the OVA-C liposomes to elicit a significant humoral immune response to vaccination compared to that seen in mice vaccinated with free OVA-C.

#### 2.3.2. T Cell Response to Vaccination

**Figure 4:**
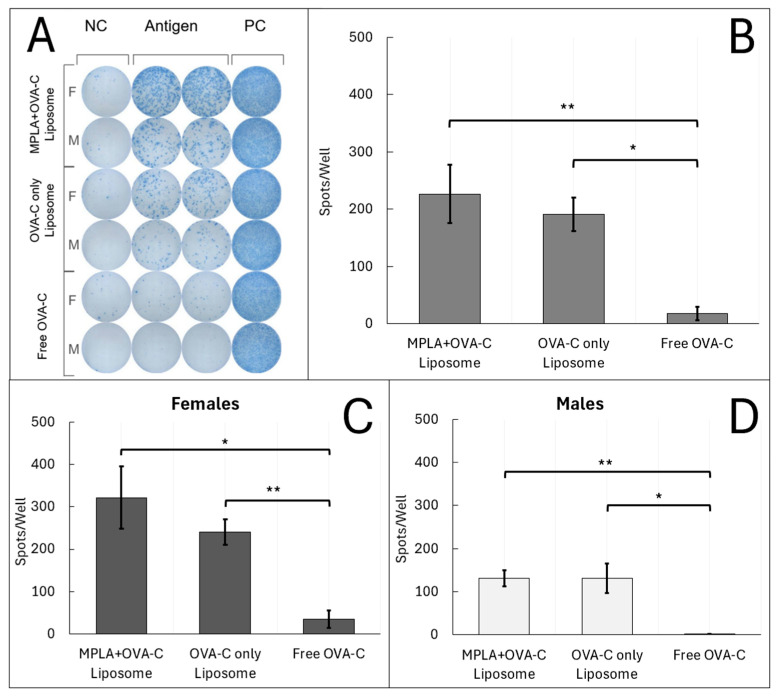
ELISpot Analysis: Liposomes induce IFN-γ T cell response to OVA-C. Mice were vaccinated with MPLA + OVA-C liposomes, OVA-C-only liposomes, or free OVA-C peptide, and tissue was collected after two weeks. Splenocytes from vaccinated mice were plated at 300 k cells/well and incubated for 24 h with either media alone (NC), media + 6 μg of OVA-C (Antigen), or media + cell proliferation cocktail (PC). (**A**) Wells were developed using a modified ImmunoSpot protocol and counted. The scanned plate is representative of the 4 cohorts that were run. (**B**–**D**) Spot counts in the representative scanned plate are reported as the average number of spots in antigen wells—NC wells. Error bars represent standard error. The experiment was performed with 5 cohorts; *n* = 10–12 per vaccination group (* *p* < 0.05) (** *p* < 0.01).

Mice vaccinated with OVA-C liposomes, with or without MPLA, displayed a more than 10-fold increase in the number of T cells producing IFN-γ in response to OVA-C vaccination as compared to the response in mice vaccinated with free OVA-C, as shown using ELISpot analysis (Figure 4). When sex was aggregated, mice vaccinated with free OVA-C showed an average of 17 ±11 IFN-γ spots per well (mean ± standard error). In contrast, mice vaccinated with OVA-C-only liposomes had an average of 191 ± 29 spots per well (*p* = 0.0001 compared to free OVA-C), and those vaccinated with MPLA + OVA-C liposomes had an average of 227 ± 51 spots per well (*p* = 0.003 compared to free OVA-C) (Figure 4B).

Unlike the humoral response, sex-based differences in the T cell response were less pronounced and were not significantly different between females and males within any vaccination group. Overall, these findings indicate that vaccination with liposomal formulations of OVA-C, regardless of MPLA inclusion, resulted in a robust cellular immune response, with an approximately 10-fold greater number of antigen-specific T cells secreting IFN-γ compared to vaccination with free OVA-C peptide.

## 3. Discussion

The results of this study demonstrate the ability of C3-liposomes to bind antigens to external OPSS groups, to target APCs via an endogenous complement-dependent mechanism, and to elicit a robust adaptive immune response. An HPLC analysis confirmed the efficient binding and retention of OVA-C peptide on the liposome surface. Importantly, this covalent binding competed with, but did not completely block, subsequent complement C3 binding. This result indicates that OPSS functional groups on C3-liposomes can support the disulfide conjugation of both the antigen and complement, leaving the interior of the liposome free for the delivery of any drug or package (i.e., protein or nucleic acid) of interest. Furthermore, these experiments demonstrate the flexibility of the system for the conjugation of hydrophilic molecules bearing a sulfhydryl group to the exterior of OPSS-liposomes while still retaining targeted uptake by APCs.

In vivo biodistribution experiments demonstrated that the C3-mediated targeting mechanism remains active in both the blood and spleen despite partial displacement of the complement corona by the antigen peptide. Interestingly, uptake into splenic myeloid cells differed from previous findings [9], in that control liposomes lacking OPSS exhibited higher-than-expected uptake. Several factors could account for this discrepancy. In these reported experiments, liposomes were only incubated for 1 h, compared to 4 h in prior experiments. Extended incubation time could lead to more pronounced differences in uptake between C3-targeted and control liposomes, as previously reported. Additionally, changes in the lipid composition may have increased non-specific uptake through a complement-independent mechanism. Lipid composition is known to strongly influence liposome biodistribution [15], suggesting that the further optimization of lipid formulation and time points is warranted to enhance uptake specificity.

OVA-C-conjugated liposomes generated significantly stronger adaptive immune responses than free OVA-C peptide, which is poorly immunogenic. Both cellular and humoral responses were markedly enhanced in mice vaccinated with OVA-C liposomes. The inclusion of MPLA in the liposomes further enhanced the IgG antibody response, although it had a limited effect on T cell activation. MPLA was selected as an adjuvant due to its hydrophobic nature, allowing for incorporation into liposomal membranes, and for its known ability to activate TLR4 signaling pathways. TLR4 activation plays a crucial role in APC maturation, antigen presentation, and T cell differentiation and maturation [16]. While MPLA effectively boosted humoral responses, alternative adjuvants targeting TLR9 (e.g., CpG) or TLR3 (e.g., poly I:C) may differentially modulate the adaptive response, potentially enhancing cellular immunity through distinct signaling cascades.

Sex-based differences in immune responses to vaccination were observed, with female mice having stronger adaptive responses overall. Statistically significant differences were observed only in the humoral responses of female mice vaccinated with MPLA + OVA-C liposomes compared to their male counterparts. Among males, the addition of MPLA did not significantly alter antibody concentrations. Interestingly, while the addition of MPLA in the vaccination of female mice increased antibody concentrations, it also increased variability between mice. In females, MPLA more than doubled the standard error in both ELISpot and ELSA results, suggesting heterogeneous responses within this group. In contrast, MPLA reduced variability in T cell responses among male mice. This MPLA-associated increase in immune variability among females may stem from sex-specific differences in TLR4 signaling and hormone regulation. Other studies have shown mixed data on TLR4 expression and antigen presentation in macrophages between male and female mice, with some data suggesting a consistently greater expression of TLR4 in female-derived macrophages [17] and some studies suggesting no difference, or even greater sensitivity to LPS in male-derived macrophages [18]. Additional data suggest that when a TLR4 agonist is used in combination with TLR7/8 and TLR9 agonists, sex differences in IgG responses to a vaccine may be reduced compared to vaccination with a TLR7/8 agonist alone [12]. A recent review by Popotas [19] showed that estrogen and progesterone levels can modulate TLR4 expression throughout the estrous cycle. Indeed, the activation of estrogen receptor alpha has been demonstrated to inhibit TLR4 signaling [20] in murine macrophages, and estrogen receptor elements have been found within the promoter for murine TLR4 [21], inhibiting the expression of TLR4. Given that the female mice in our experiments were collected in multiple, separate cohorts, without the synchronization of estrus stages, hormonal fluctuations may partially explain the increased variance observed in female immune responses. Further study of sex differences in TLR agonist signaling is needed to corroborate these findings and examine the increased sensitivity to MPLA in female mice. The myeloid-targeting ability of our C3-liposomal system may be a useful tool for future in vivo experiments.

Some of the manufacturing challenges of nanoparticle-based vaccines include the stability of the nanoparticle formulation, the cost of peptides and proteins, and the difficulty of purifying the nanoparticles from unconjugated components when scaling up to pharmaceutically relevant quantities. Our present system addresses these difficulties first by greatly increasing the conjugation efficiency from 5% with passive encapsulation to 84.6% with the strategy of disulfide-binding peptide to the exterior of the liposomes [12]. This level of conjugation will greatly decrease the quantity of peptide needed and could avoid a purification step, as the vast majority of the peptide is bound to the liposomes. In addition, the liposome formulation showed impressive stability over 15 months with no visible aggregation, a modest increase in liposome size from 255 ± 98 nm to 368 ± 124 nm, and a retained ability to internalize in vitro, as demonstrated by uptake into bone marrow myeloid cells (Figure S1).

In conclusion, this study demonstrates the effectiveness of a novel liposomal delivery platform targeting myeloid cells and eliciting both potent T cell and B cell responses in mice. The incorporation of MPLA adjuvant into the C3-liposomes significantly enhanced the humoral immune response but also revealed sex-based differences in immunogenicity, highlighting the importance of considering sex as a biological variable in future research. The modular design of our liposomal system allows for the flexible, targeted delivery of single or multiple antigens, making it a promising tool for vaccine development in cancer and other disease applications. Furthermore, the ability to rapidly synthesize and conjugate custom peptides to the exterior of our complement C3-targeted liposomes makes this delivery system potentially useful in future scenarios, such as for vaccines in emerging epidemics with rapidly evolving organisms or for personalized cancer immunotherapy targeting identified neoantigens in rapidly mutating tumors. Although the scope of this work did not allow for testing the immune response to tumor antigens, future studies will explore the liposome delivery system using tumor antigens such as TRP2 in B16F10 melanomas to treat mouse models of cancer.

## 4. Materials and Methods

### 4.1. Materials

1,2-dipalmitoyl-sn-glycero-3-phosphocholine (DPPC), 1,2-distearoyl-sn-glycero-3-phosphocholine (DSPC), and 1,2-distearoyl-sn-glycero-3-phosphoethanolamine-N-[PDP-poly(ethylene glycol)-2000] (DSPE-PEG(2000)-PDP) for liposome preparation were purchased from Avanti Polar Lipids (Alabaster, AL, USA). As with previous publications, we refer to the PDP group as OPSS [9]. Size exclusion chromatography was performed using CL4B Sepharose gel (Sigma-Aldrich, St. Louis, MO, USA). Fluorescently tagged lipid, lissamine rhodamine B 1,2-dihexadecanoyl-sn-glycero-3-phosphoethanolamine (Rhodamine-PE), was purchased from Life Technologies (Grand Island, NY, USA). Monophosphoryl Lipid A (MPLA) was purchased from InvivoGen (San Diego, CA, USA). The peptide sequence ISQAVHAAHAEINEAGRGGGC was synthesized by Genscript (Piscataway, NJ, USA). CL4B Sepharose gel used in the size exclusion chromatography of liposomes was purchased from Sigma-Aldrich (St. Louis, MO, USA). Alexafluor700 CD11b antibody was purchased from BioLegend (San Diego, CA, USA). For the ELISA, 96-well flat-bottom Maxisorp plates, streptavidin, and SMPH (succinimidyl 6-((beta-maleimidopropionamido)hexanoate)) were ordered from Thermo Scientific (Pittsburgh, PA, USA). Goat anti-murine IgG-horseradish peroxidase (HRP), used as the secondary antibody, was ordered from Abcam (ab6789) (Waltham, MA, USA). Murine anti-OVA IgG, used as a standard, was ordered from Sigma-Aldrich (St. Louis, MO, USA). Mouse IFN-γ Single-Color ELISpot kits were purchased from ImmunoSpot (Cleveland, OH, USA). Mouse serum with functional complement was ordered from Innovative Research (Novi, MI, USA). All other chemicals and reagents were purchased from Thermo Fisher Scientific (Pittsburgh, PA, USA).

### 4.2. Liposome Preparation

Liposomes were prepared from lipid stocks suspended in chloroform. DSPC:DPPC:DSPE–rhodamine:MPLA:DSPE-PEG(2000)-PDP (DSPE-PEG(2000)-OPSS) were mixed at a molar ratio of 21:72:1:1:5 for MPLA+ liposomes and without MPLA for liposomes that did not contain the adjuvant. Control liposomes had a similar composition except with DSPE-PEG(2000) substituted for DSPE-PEG(2000)-OPSS. The lipids were mixed and dried at 47 °C under gaseous nitrogen for one hour and then further dried under vacuum at room temperature (RT) for two hours to remove any remaining chloroform. The lipid film was hydrated in 0.7 mL of water at 47 °C and extruded through a 400 nm polycarbonate membrane filter 9 times (Avestin, Ottawa, ON, Canada). After extrusion, liposomes were incubated overnight at RT with a custom peptide ISQAVHAAHAEINEAGRGGGC (OVA-C) synthesized by Genscript. In total, 25 μL of 1.2 mg/mL peptide in 1× PBS (pH 7.4) OVA-C was added to 200 μL of liposomes to obtain a final concentration of 133 μg/mL.

Liposome-bound antigen concentration was quantified using HPLC. Liposomes and free OVA-C vaccinations were normalized to a concentration of 35 μg/mL OVA-C. Liposome conjugation efficiency was calculated by the following equation:

[fraction volume off CL4B Sepharose column] × [conc. of OVA-C conjugated to liposomes] / [total OVA-C added to liposomes] = % conjugation efficiency

For in vitro experiments, following the CL4B column, liposomes were incubated for 30 min at RT with 10% *v*/*v* C3+ C57BL/6 mouse serum. Following antigen and complement C3 incubation, the liposomes were column-purified using a CL4B Sepharose column hydrated in 1× PBS and collected in a 0.7 mL fraction. Resulting liposomes were analyzed by HPLC and normalized to 35 μg/mL OVA-C. In vivo experiments relied on endogenous complement.

### 4.3. HPLC Analysis

C3-liposomes with conjugated OVA-C were analyzed using a 1200 Infinity series liquid chromatography system with diode array detection controlled by MassHunter v. B.06.00 (Agilent, Santa Clara, CA, USA). First, 100 µL aliquots of liposomes were incubated with 1 µL 1M dithiothreitol for 30 min at room temperature. Then, 10 µL injections were run on a Zorbax 300SB-C8 column (100 × 2.1 mm, 3.5 µm) (Agilent, Santa Clara, CA, USA). The column temperature was maintained at 35 °C for the method. OVA-C was eluted using a gradient method with solvent A consisting of water and solvent B consisting of acetonitrile, both containing 0.1% trifluoroacetic acid, using a flow rate of 0.3 mL/min. The gradient increased the proportion of acetonitrile from 10% to 95% over a 10-minute period, followed by a 3-minute hold before returning to the starting conditions. OVA-C was eluted at 5.48 min, the primary complement C3 peak occurred at approximately 8.39 min, and the total method run time was 20 min. Detection of OVA-C was performed using UV absorbance at 220 nm with a reference wavelength of 240 nm, and the corresponding bandwidths were 4 nm and 20 nm.

### 4.4. Biodistribution

C57BL/6 mice were intravenously injected (lateral caudal vein) with 100 μL of either MPLA + OVA-C liposomes, OVA-C-only liposomes, or control liposomes (no OPSS or MPLA), each containing rhodamine. After one hour, mice were sacrificed, and blood and spleen were collected. Blood was collected with a heparinized syringe and incubated with Red Blood Cell lysis buffer at room temperature for 5 min before PBS was added to stop the lysis. The tube was centrifuged at 420 g for 5 min, the supernatant was discarded, and the remaining cells were resuspended in 10 mL of 1× PBS. Spleens were incubated in 1 mL of 1 mg/mL collagenase for 10 min at 37 °C, 5% CO_2_. The collagenase was deactivated by adding 10 mL of RPMI 1640 + 10% FBS + 1% penicillin–streptomycin. Spleen cells were then filtered through a 100 μm cell strainer, centrifuged at 420 g for 5 min, and resuspended in 10 mL of 1× PBS. Blood and spleen cells were imaged with a Leica DMI6000B inverted fluorescence microscope using a 20× objective (Leica Microsystems, Buffalo Grove, IL, USA). Photos were taken using Leica Application Suite, version 3.7.0 (Leica Microsystems Inc., Wetzlar, Germany).

### 4.5. Flow Cytometry

Flow cytometry was performed to determine which cells had internalized rhodamine-labeled liposomes. Cells were plated in a 96-well V-bottom polystyrene microplate and centrifuged for 5 min at 420 g at 4 °C in a Sorvall Legend X1R centrifuge. After centrifugation, the cells were resuspended in 100 μL of FACS buffer (1× PBS +1% bovine serum albumin) that contained antibodies against CD45 and CD11b. Cells were incubated with the antibodies for 10 min at 21 °C in the dark to allow for conjugation before pelleting by centrifugation, as described above. The cells were then resuspended in 120 μL of FACS buffer before analysis by flow cytometry, using a Beckman Coulter CytoFLEX flow cytometer with the CytExpert software version 2.0.0.153 (Beckman Coulter, Brea, CA, USA). Myeloid cells were identified as CD45^+^ and CD11b^+^, and the percentage of myeloid cells that had internalized liposomes was determined by gating for the rhodamine label. Data are presented as mean ± standard error (*n* = 6).

### 4.6. Vaccination

Six cohorts of C57BL/6 mice were injected subcutaneously with 100 μL of either free OVA-C, OVA-C liposomes, or MPLA + OVA-C liposomes at days 0 and 7 of the 14-day vaccination experiments. In total, 4 cohorts consisted of 3 male and 3 female mice, with 1 male and 1 female per vaccination group; 2 additional cohorts had 8 and 5 mice. The ELISpot plate was overdeveloped in the 5-mouse cohort and was unusable; however, the ELISA data from the same cohort were retained.

### 4.7. Tissue Preparation for ELISA and ELISPOT

Mice were sacrificed, and whole blood and spleens were harvested. Blood was centrifuged at 420 g for 7 min to collect serum. Spleens were treated as above in the biodistribution studies but were resuspended in 10 mL of RPMI 1640 + 10% FBS + 1% penicillin–streptomycin instead of PBS for ELISpot analysis.

### 4.8. ELISA

Wells in a 96-well flat-bottom plate (MaxiSorp Thermo Scientific) were incubated with 500 ng of streptavidin (Thermo Scientific) for 1 h at 21 °C to coat wells. After coating, 500 ng of SMPH (succinimidyl 6-((beta-maleimidopropionamido)hexanoate)) was added to the wells and incubated for 1 h at 21 °C. In total, 500 ng of OVA-C peptide (ISQAVHAAHAEINEAGRGGGC) was then added to the wells and incubated for two hours at 21 °C. Wells were blocked for 1 h with 100 μL of PBS + 0.5% milk at 21 °C, after which, primary antibody or serum from the experimental mice diluted to 1:40 was added to the wells and incubated at 4 °C overnight. Murine anti-OVA IgG (Sigma-Aldrich) was used to produce a standard curve, and a 3-parameter exponential curve was used to fit. One female MPLA + OVA-C sample with an optical density beyond the highest standard concentration of the standard curve was recorded as the highest standard concentration. Horseradish HRP-goat anti-murine IgG was used as a secondary antibody, diluted 1:4000 in 0.5% PBS milk. A volume of 100 μL of TMB was added as substrate for the enzyme and was halted by the addition of 1% HCl, and the plate absorbance was analyzed at 450 nm. This method was developed and kindly shared by Dr. Alex Francian [14].

### 4.9. ELISpot

Spleen cells were plated in ImmunoSpot anti-murine IFN-γ strips at 300,000 cells per well in 200 μL of RPMI 1640 +10% FBS + 1% penicillin–streptomycin. The wells contained either media alone (negative control), 6 μg of OVA-C (antigen), or PMA (positive control). Cells were incubated for 24 h at 37 °C and 9% CO_2_ before the wells were developed according to ImmunoSpot’s protocol. Spots were counted using an ImunoSpot S6 Micro analyzer (ImmunoSpot, Shaker Heights, OH, USA). Quality control included negative controls to ascertain baseline IFN-y release of each sample, positive controls to determine cell and plate development viability, and duplicate wells at varying cell densities to determine appropriate paracrine stimulation. Negative control well counts were subtracted from the average of the duplicate antigen well counts.

### 4.10. Statistical Analysis

Statistical analyses were performed by JMP Pro 17 and R (version 4.4.2) [22]. Normalcy was tested using the Shapiro–Wilk test. An unpaired two-tailed *t*-test assuming unequal variance was used to compare normally distributed datasets, while a nonparametric comparison (Wilcoxon method) was used for nonparametric datasets. Alpha values of less than 0.05 were considered significant and are indicated by an asterisk, while alpha values of less than 0.01 are indicated by two asterisks. Figure 2 was generated using *ggplot2* [23], *ggpubr* [24], and *cowplot* [25].

## Data Availability

Data is contained within the article and Supplementary Materials.

## Supplementary Materials

The following supporting information can be downloaded at https://www.mdpi.com/article/10.3390/ijms26114985/s1.

## Abbreviations

The following abbreviations are used in this manuscript:

APCs	Antigen-presenting cells
C3	Complement protein 3
CR3	Complement receptor 3
OVA	Ovalbumin
OVA-C	Ovalbumin peptide with -GGGC tag
TLR	Toll-like receptor
MPLA	Monophosphoryl lipid A
CTL	Cytotoxic T lymphocyte
DCs	Dendritic cells
OPSS	Orthopyridyl disulfide group
SMPH	Sulfopropyl-N-hydroxysuccinimide
