# Supplementary material for: Immunotherapy Platform That Conjugates Antigen to Complement C3-Targeted Liposomes Induces a Robust Adaptive Immune Response
Source: Int J Mol Sci. 2025 May 22;26(11):4985. doi: 10.3390/ijms26114985 (PMC12155296; doi:10.3390/ijms26114985)
Supplement: Supplementary file 1 [file ijms-26-04985-s001.zip › ijms-3620994-supplementary.pdf]

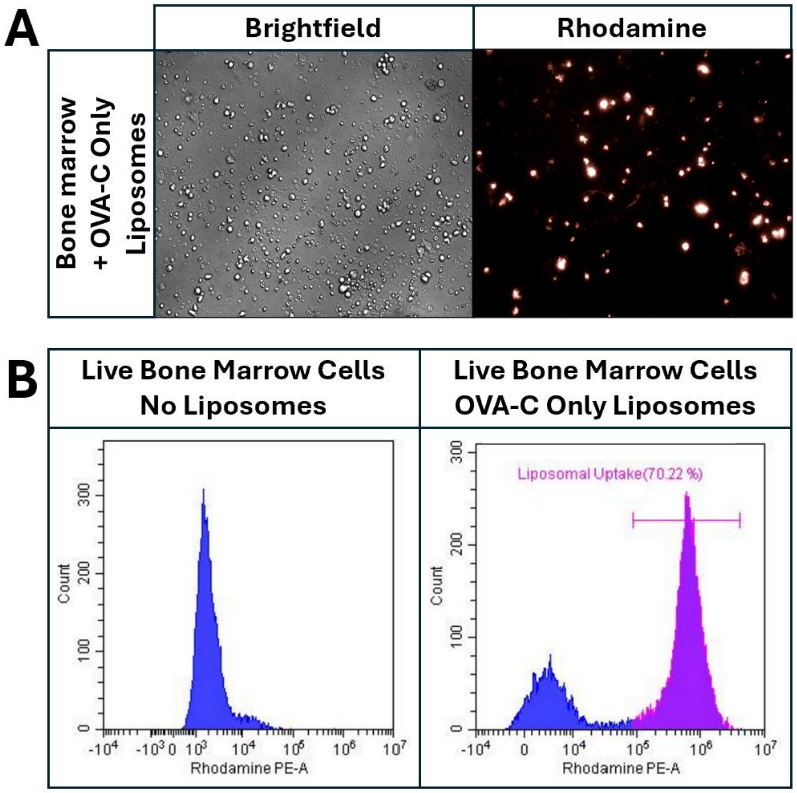

Supplementary Figure 1: OVA-C bearing liposomes retain C3 mediated uptake in vitro in bone marrow cells after 15 months at 4°C. Rhodamine containing OVA-C only liposomes used in 2024 vaccination experiments, and stored for 15 months, were incubated with C3+ murine serum for 30 minutes at room temperature. Murine bone marrow cells were collected from the femur and tibia of Balb/c mice, incubated in RPMI 1640 + 10% FBS + 1% penicillin-streptomycin with and without liposomes for 2 hours at 37 ° C and 5% CO_2 and analyzed as described in the flow cytometry and biodistribution sections of the materials and methods, with fluorescent microscopy images taken using a 20x objective. (A) Cells were imaged via fluorescence microscopy. (B) Cells were analyzed by flow cytometry for rhodamine fluorescence after singlet and live cell gating, showing that 70.22% of cells had taken up liposomes.
